# A novel mean-centering method for normalizing microRNA expression from high-throughput RT-qPCR data

**DOI:** 10.1186/1756-0500-4-555

**Published:** 2011-12-21

**Authors:** Dennis Wylie, Jeffrey Shelton, Ashish Choudhary, Alex T Adai

**Affiliations:** 1 Asuragen Inc, Austin, TX 78744, USA

## Abstract

**Background:**

Normalization is critical for accurate gene expression analysis. A significant challenge in the quantitation of gene expression from biofluids samples is the inability to quantify RNA concentration prior to analysis, underscoring the need for robust normalization tools for this sample type. In this investigation, we evaluated various methods of normalization to determine the optimal approach for quantifying microRNA (miRNA) expression from biofluids and tissue samples when using the TaqMan^® ^Megaplex™ high-throughput RT-qPCR platform with low RNA inputs.

**Findings:**

We compared seven normalization methods in the analysis of variation of miRNA expression from biofluid and tissue samples. We developed a novel variant of the common mean-centering normalization strategy, herein referred to as mean-centering restricted (MCR) normalization, which is adapted to the TaqMan Megaplex RT-qPCR platform, but is likely applicable to other high-throughput RT-qPCR-based platforms. Our results indicate that MCR normalization performs comparable to or better than both standard mean-centering and other normalization methods. We also propose an extension of this method to be used when migrating biomarker signatures from Megaplex to singleplex RT-qPCR platforms, based on the identification of a small number of normalizer miRNAs that closely track the mean of expressed miRNAs.

**Conclusions:**

We developed the MCR method for normalizing miRNA expression from biofluids samples when using the TaqMan Megaplex RT-qPCR platform. Our results suggest that normalization based on the mean of all fully observed (fully detected) miRNAs minimizes technical variance in normalized expression values, and that a small number of normalizer miRNAs can be selected when migrating from Megaplex to singleplex assays. In our study, we find that normalization methods that focus on a restricted set of miRNAs tend to perform better than methods that focus on all miRNAs, including those with non-determined (missing) values. This methodology will likely be most relevant for studies in which a significant number of miRNAs are not detected.

## Background

MicroRNAs (miRNAs) are small, non-coding RNA molecules that have key regulatory roles in mammalian cells. Misregulation of miRNA expression has been implicated in several diseases including cancers, resulting in a growing interest in identifying miRNA biomarkers with diagnostic value [1]. Extracellular circulating miRNAs have been detected in serum, plasma, and other body fluids (collectively referred to as biofluids) by real-time quantitative RT-PCR (RT-qPCR), opening up the possibility for the use of these miRNAs as novel clinical biomarkers [2,3].

TaqMan Megaplex RT-qPCR technology enables the simultaneous detection of 377 miRNAs from a single reverse transcription reaction, greatly reducing the amount of starting material and the number of RT reactions required for quantitative gene expression analysis [4]. Because singleplex RT-qPCR is considered to have the highest standard of sensitivity and is a preferred format for clinical tests, it will be critical to develop analytical tools to reconcile differences between the two platforms, so that potential miRNA biomarkers can be more accurately migrated to development.

Normalization--the process of reducing technical error or variation between samples--is critical for accurate expression analysis. In studies with tissue samples, RNA input is typically equalized between samples before analysis, removing variation due to RNA concentration differences. One of the most significant challenges in quantifying miRNA expression from biofluids is the fact that the RNA concentration is typically below the limit of quantitation by spectrophotometry, making it difficult to measure and equalize RNA input levels before analysis by RT-qPCR. Another challenge is the lack of standardized protocols for RNA purification from biofluids; further increasing variability when comparing samples that have undergone different procedures.

Normalization of singleplex miRNA RT-qPCR data from solid tissue samples has been thoroughly evaluated at Asuragen [5] and elsewhere [6-8]. However, our current work is focused on the high-throughput analysis of miRNA expression using the TaqMan Megaplex RT-qPCR platform. Thus, we propose MCR, a novel variation on the strategy of mean-centering, for normalizing miRNA RT-qPCR data when using the TaqMan Megaplex platform. We also propose CCR, a normalizer selection strategy to enable migrating signatures from Megaplex to singleplex RT-qPCR. This strategy aims to identify normalizer miRNAs whose expression values across samples most closely track the mean value of all miRNAs.

## Materials and methods

### RNA isolation and RT-qPCR

All experimental work was performed at Asuragen by Asuragen's Pharmacogenomics Services Group using internally developed and optimized protocols. Human placenta and brain total RNA was obtained from Ambion, part of Life Technologies. For these reference RNAs, six different mass inputs (100 ng, 10 ng, 1 ng, 0.5 ng, 0.05 ng, and 0.005 ng) were used for reverse transcription (RT). For the biofluid studies, blood samples were collected from healthy donors after obtaining informed consent, under institutional review board-approved protocols. Sera was pooled, divided into aliquots, and stored at -80°C. Total RNA was isolated from the serum and the RNA equivalents of serum volumes of 300 μL, 200 μL, 100 μL, 50 μL, and 25 μL were used for RT. TaqMan Megaplex RT and preamplification reactions were performed using equal volumes of input RNA according to the manufacturer's protocol, and real-time PCR was run on the TaqMan miRNA Array Card A using the Applied Biosystems 7900HT Real-Time PCR System. Data were processed and exported with Applied Biosystems SDSv2.3 software, and were subsequently analyzed using the R programming language.

### Normalization

Additional file 1: Figure S1 and Additional file 1: Figure S2 depict the pseudocode for the MCR and CCR algorithms, respectively. Existing normalization methods were implemented as described previously [6-11] or are available through the appropriate R packages (limma for MAD-scaling and quantile normalization, and epiR for the estimate of the concordance correlation coefficient). The geNorm and NormFinder algorithms were implemented at Asuragen, and were run to choose normalizers considering only fully detected miRNAs (those with Ct values less than 40). Normalizer selection using the CCR algorithm was also restricted to fully detected miRNAs, though for this algorithm a Ct threshold of less than 35 was required for a miRNA to be considered detected in a given sample.

It should be noted that the presence of one or more samples with significantly lower overall RNA content can significantly reduce the size of the fully detected miRNA set. This problem, particularly for normalizer-based methods, is compounded by the increased noise in Ct determination for probes detected only at very high Ct values (35-40 Ct), which may render the measurements for such probes unreliable. As a result, the CCR algorithm provides an option to treat Ct values above a user-specified threshold as non-detected. This threshold may be adjusted upward in the presence of RNA-depleted samples, to include miRNAs that are detected strongly in most samples but weakly in samples with depleted RNA content. Alternately, the maximum Ct threshold of the CCR algorithm may be adjusted downward when no such depleted samples are present, thereby removing weakly detected miRNAs.

## Results and discussion

We evaluated methods that were previously developed for normalization of RT-qPCR messenger RNA (mRNA) data. These methods include geNorm [6] and NormFinder [7], alongside the conceptually simpler mean-centering (MC) method proposed by Mestdagh et al. [8]. We also considered two other techniques developed for normalization of mRNA microarray data: the median absolute deviation (MAD) scaling algorithm (Scale) [9,10], and quantile normalization [11]. Furthermore, we developed and investigated two additional strategies of normalizing miRNA RT-qPCR data when using the TaqMan Megaplex platform. The first method extends the advantages of mean-centering normalization [8] to situations in which the mean itself may be unreliable, e.g., in biofluid miRNA RT-qPCR samples for which a substantial fraction of miRNA data values may be missing. This approach, which we refer to as mean-centering restricted (MCR), is designed to track the mean of only the miRNAs found present (100% detected) across all samples (See Additional file 1: Figure S1 for pseudocode). As an extension of this method, we also developed a normalization strategy that will be applicable when migrating from Megaplex (hundreds of miRNAs) to singleplex (generally tens of miRNAs) RT-qPCR. This strategy, herein referred to as concordance correlation restricted (CCR) normalization, uses a concordance correlation coefficient [12] to select miRNAs that are concordant with the restricted mean expression value (See Additional file 1: Figure S2 for pseudocode).

**Figure 1:**
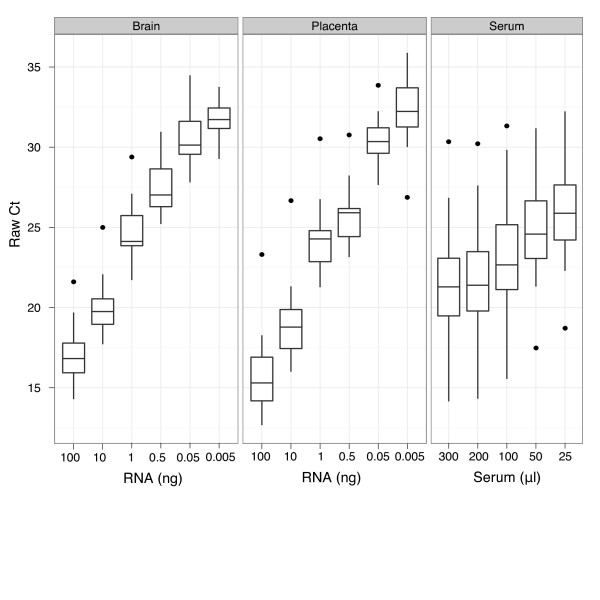
**Distribution of Ct values of unnormalized RT-qPCR data**. The distribution of raw (unnormalized) Ct values are shown for each titration series performed with brain, placenta, and serum samples. The percent non-determined calls (Ct > 40) significantly increased with the mean Ct value of the sample.

**Figure 2:**
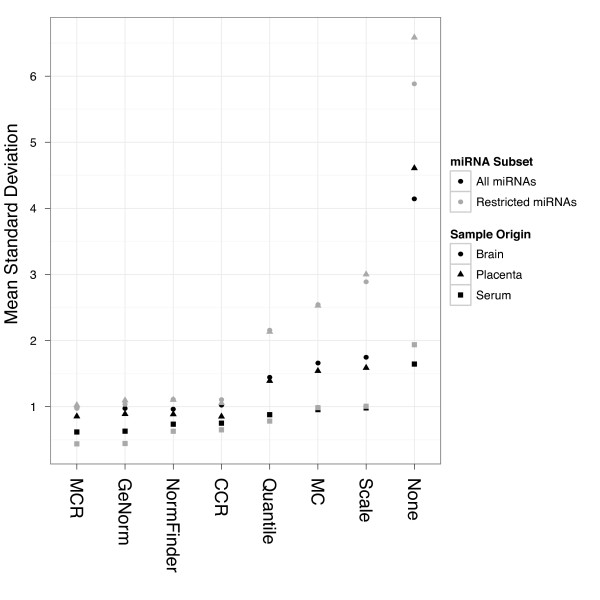
**Effect of normalization method on variation of miRNA expression**. Each point represents the mean standard deviation from all miRNAs (All miRNAs; n = 377) or the restricted set of miRNAs (Restricted miRNAs; n = 19) on the TaqMan array, but calculated separately across all samples within a given group (Sample Origin). The restricted set of miRNAs is the core set of miRNAs detected across all samples in all titrations. Note that all data were normalized together, and this is most important for methods that share information across samples. NormFinder was parameterized to use the sample origin for grouping. GeNorm, NormFinder, and CCR results are based on the selection of two miRNAs as normalizers.

We used titration studies (Figure 1) to evaluate each normalization method on Megaplex RT-qPCR data. In each study, we calculated the standard deviations of normalized expression levels of each miRNA associated with each normalization method (Figure 2 and Additional file 1: Figure S3). We found the MCR method to produce among the lowest mean estimates of standard deviations compared to the other normalization methods. The normalizer-based methods (geNorm, NormFinder, and CCR), which subtract the mean expression values of a given subset of miRNAs from all other miRNAs, also performed well by this metric; on the other hand, MAD-scaling and quantile normalization joined non-restricted mean-centering (MC) in showing relatively poor performance with regard to minimization of technical variance.

**Figure 3:**
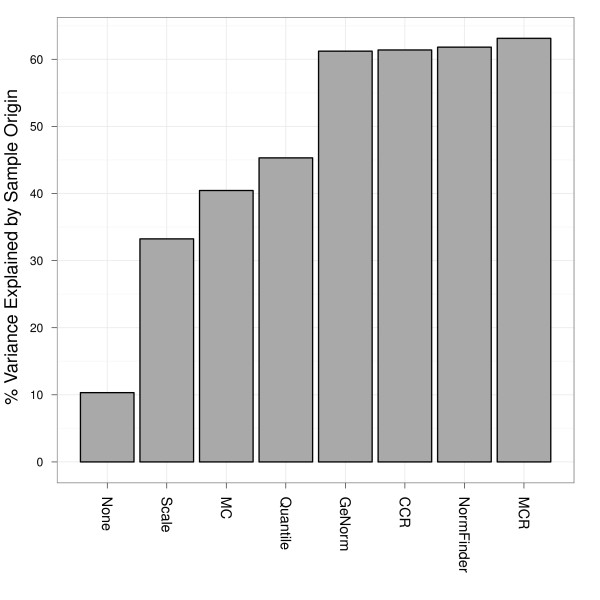
**Variance explained by sample origin**. Bars show the percent variance explained by sample origin (tissue type) based on weighting results from a univariate random effect model using the eigenvalues from principal component analysis (PCA). We used the first three principal components and their corresponding eigenvalues for weighting (See reference [13] for more information). In general, MCR normalization tends to reveal more of the biological differences between samples, and shows nominal improvement over other miRNA (gene)-specific normalization methods.

Next, we determined which normalization procedure best captured the underlying biological differences (i.e., the tissue origin) between samples. We used variance principal component analysis [13] to estimate the percent variance explained by the biological (tissue) origin of the samples used in the titration studies (Figure 3). The results suggest that MCR normalization and the normalizer-based methods are better able to capture the tissue origin of the samples compared to the other normalization methods (MAD-scaling, quantile normalization, and MC).

The CCR normalizers selected from the titration studies were miR-222 and miR-320; the average value of these two miRNAs had a concordance correlation coefficient with the restricted sample means of 0.992 (95% CI: 0.978-0.997), while individually the two had concordance correlations of 0.986 (miR-222) and 0.971 (miR-320) with the restricted sample mean (the median concordance correlation for all miRNAs with the restricted sample mean was 0.504, while the median Pearson correlation was 0.851). We specified the output of two normalizers from all three normalizer algorithms (geNorm, NormFinder, and CCR) applied to the titration study to facilitate algorithmic comparisons, but it is worth noting that the improvement in concordance correlation from adding the second normalizer is relatively small - if we had used the convergence criterion (See Materials and Methods) of our implementation of CCR instead of specifying the normalizer count, only miR-222 would have been selected. This is likely attributable to the high level of correlation between the Ct measurements of different miRNAs over a data set whose variance is largely driven by overall input RNA amount. While most data sets of interest will have considerably less variation in input RNA compared to a titration study, we have regularly seen CCR produce only 1 or 2 normalizer candidates.

The results from both experimental approaches suggest that the MCR normalization method performs comparable to or better than the other methods according to the standard deviation metric. Additionally, the CCR method exhibits comparable performance to the more established normalizer-based methods, geNorm and NormFinder. The CCR method will be applicable when assays are migrated from multiplex to singleplex RT-qPCR platforms. We note that the algorithms with strong performance (MCR, CCR, geNorm, and NormFinder) have one major feature in common: all analyze only fully detected miRNAs. However, NormFinder and geNorm implement complex algorithms for identifying normalizers. In contrast, the MCR and CCR algorithms are conceptually simpler because they rely only on a mean estimate of miRNA expression; thus, they are likely to be more practical to implement, especially for larger datasets. For example, our implementation of CCR ran more than 100 times faster than either NormFinder or geNorm when applied to a randomly generated matrix of 500 pseudogenes (with no missing values) and 20 pseudosamples (0.13 s for CCR, 29 s for NormFinder, and 2700 s for geNorm) (data not shown).

## Conclusion

There is a critical need for robust methods of normalizing miRNA expression data from biofluid samples and other sample types with low RNA inputs. In general, we observed that normalizing the data is beneficial compared to the absence of normalization, and that array-based normalization methods (Scale, MC, MCR, and Quantile) tend to perform worse than miRNA (gene)-specific normalization methods (CCR, GeNorm, and NormFinder), with the exception of MCR. The MCR method, based on the mean of all fully detected miRNAs, reduced the standard deviations across the titration samples, while also showing maximum separation between true biologically different sample types using variance principal component analysis. Our results suggest that the conceptually simple MCR (and its cousin implementation CCR) normalization strategy performs comparable to or better than existing methods for normalization of high-throughput RT-qPCR data. This strategy is well suited for studies in which a significant number of expression values are missing (non-determined), including studies with biofluids samples.

Normalizer-based methods require that at least one miRNA is fully detected across all samples. In cases where no miRNAs have 100% detection, removal of the potential outlier sample(s) usually remedies the situation. The optimal normalization strategy for any given study can be exhausting to uncover, but MCR and CCR should be among the first normalization methods to evaluate. To enable MCR and CCR adoption, we have made the source code freely available (See Additional file 2). In general, we would recommend using any normalizer-based method that relies on fully detected miRNAs.

## List of abbreviations

CCR: concordance correlation restricted; MAD: median absolute deviation; MC: mean-centering; MCR: mean-centering restricted; miRNA: microRNA; PCA: principal component analysis; RT-qPCR: real-time quantitative RT-PCR.

## Competing interests

The authors declare that they have no competing interests.

## Authors' contributions

ATA and DW designed the analysis plan; DW carried out the analysis; JS performed the experimental work; AC implemented the geNorm and NormFinder algorithms; DW and ATA wrote the paper. All authors read and approved the final manuscript.

## Supplementary Material

Additional file 1 **Supplemental Figures**. This document contains 3 supplemental figures referenced in the main text. The first two supplemental figures are pseudocodes detailing the MCR and CCR algorithms, while the third figure shows the overall distribution of standard deviations after application of different normalization methods. Click here for file

Additional file 2 **R source code implementing MCR and CCR**. This file contains source code that implements the MCR and CCR algorithms. A small example that demonstrates usage is also provided. Click here for file

## References

[B1] Cortez MA Ivan C Zhou P Wu X Ivan M Calin GA microRNAs in cancer: from bench to bedside Adv Cancer Res 2010 108 113 157 21034967 10.1016/B978-0-12-380888-2.00004-2

[B2] Etheridge A Lee I Hood L Galas D Wang K Extracellular microRNA: a new source of biomarkers Mutat Res 2011 717 1-2 85 90 10.1016/j.mrfmmm.2011.03.004 21402084 PMC3199035

[B3] Kroh EM Parkin RK Mitchell PS Tewari M Analysis of circulating microRNA biomarkers in plasma and serum using quantitative reverse transcription-PCR (qRT-PCR) Methods 2010 50 298 301 10.1016/j.ymeth.2010.01.032 20146939 PMC4186708

[B4] Mestdagh P Feys T Bernard N Guenther S Chen C Speleman F Vandesompele J High-throughput stem-loop RT-qPCR miRNA expression profiling using minute amounts of input RNA Nucleic Acids Res 2008 36 e143 10.1093/nar/gkn725 18940866 PMC2588502

[B5] Peltier HJ Latham GJ Normalization of microRNA expression levels in quantitative RT-PCR assays: identification of suitable reference RNA targets in normal and cancerous human solid tissues RNA 2008 14 844 852 10.1261/rna.939908 18375788 PMC2327352

[B6] Vandesompele J De Preter K Pattyn F Poppe B Van Roy N De Paepe A Speleman F Accurate normalization of real-time quantitative RT-PCR data by geometric averaging of multiple internal control genes Genome Biol 2002 3 RESEARCH0034 12184808 10.1186/gb-2002-3-7-research0034 PMC126239

[B7] Andersen CL Jensen JL Orntoft TF Normalization of real-time quantitative reverse transcription-PCR data: a model-based variance estimation approach to identify genes suited for normalization, applied to bladder and colon cancer data sets Cancer Res 2004 64 5245 5250 10.1158/0008-5472.CAN-04-0496 15289330

[B8] Mestdagh P Van Vlierberghe P De Weer A Muth D Westermann F Speleman F Vandesompele J A novel and universal method for microRNA RT-qPCR data normalization Genome Biol 2009 10 R64 10.1186/gb-2009-10-6-r64 19531210 PMC2718498

[B9] Yang YH Dudoit S Luu P Lin DM Peng V Ngai J Speed TP Normalization for cDNA microarray data: a robust composite method addressing single and multiple slide systematic variation Nucleic Acids Res 2002 30 e15 10.1093/nar/30.4.e15 11842121 PMC100354

[B10] Smyth GK Speed T Normalization of cDNA microarray data Methods 2003 31 265 273 10.1016/S1046-2023(03)00155-5 14597310

[B11] Bolstad BM Irizarry RA Astrand M Speed TP A comparison of normalization methods for high density oligonucleotide array data based on variance and bias Bioinformatics 2003 19 185 193 10.1093/bioinformatics/19.2.185 12538238

[B12] Lin LI A concordance correlation coefficient to evaluate reproducibility Biometrics 1989 45 255 268 10.2307/2532051 2720055

[B13] Scherer A Batch effects and noise in microarray experiments: sources and solutions 2009 Chichester, U.K.: J. Wiley

