# Supplementary material for: A novel mean-centering method for normalizing microRNA expression from high-throughput RT-qPCR data
Source: BMC Res Notes. 2011 Dec 21;4:555. doi: 10.1186/1756-0500-4-555 (PMC3267743; doi:10.1186/1756-0500-4-555)
Supplement: Additional file 1 — Supplemental Figures. This document contains 3 supplemental figures referenced in the main text. The first two supplemental figures are pseudocodes detailing the MCR and CCR algorithms, while the third figure shows the overall distribution of standard deviations after application of different normalization methods. [file 1756-0500-4-555-S1.DOCX]

# Supplemental Figures

##
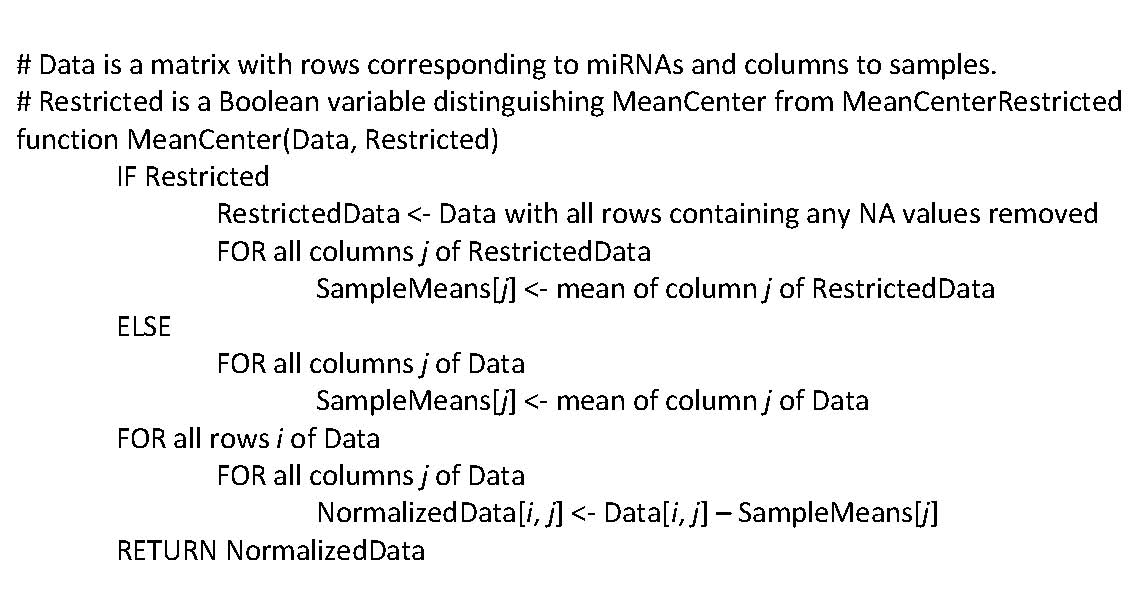


## Supplemental Figure 1 - Pseudocode for the MCR algorithm.

The text details a function, MeanCenter, that implements the MCR algorithm. The only inputs are the expression data (Data) and a Boolean switch to turn the MCR algorithm on (Restricted=TRUE) or off (Restricted=FALSE). The result of the MCR algorithm is the normalized miRNA expression values.


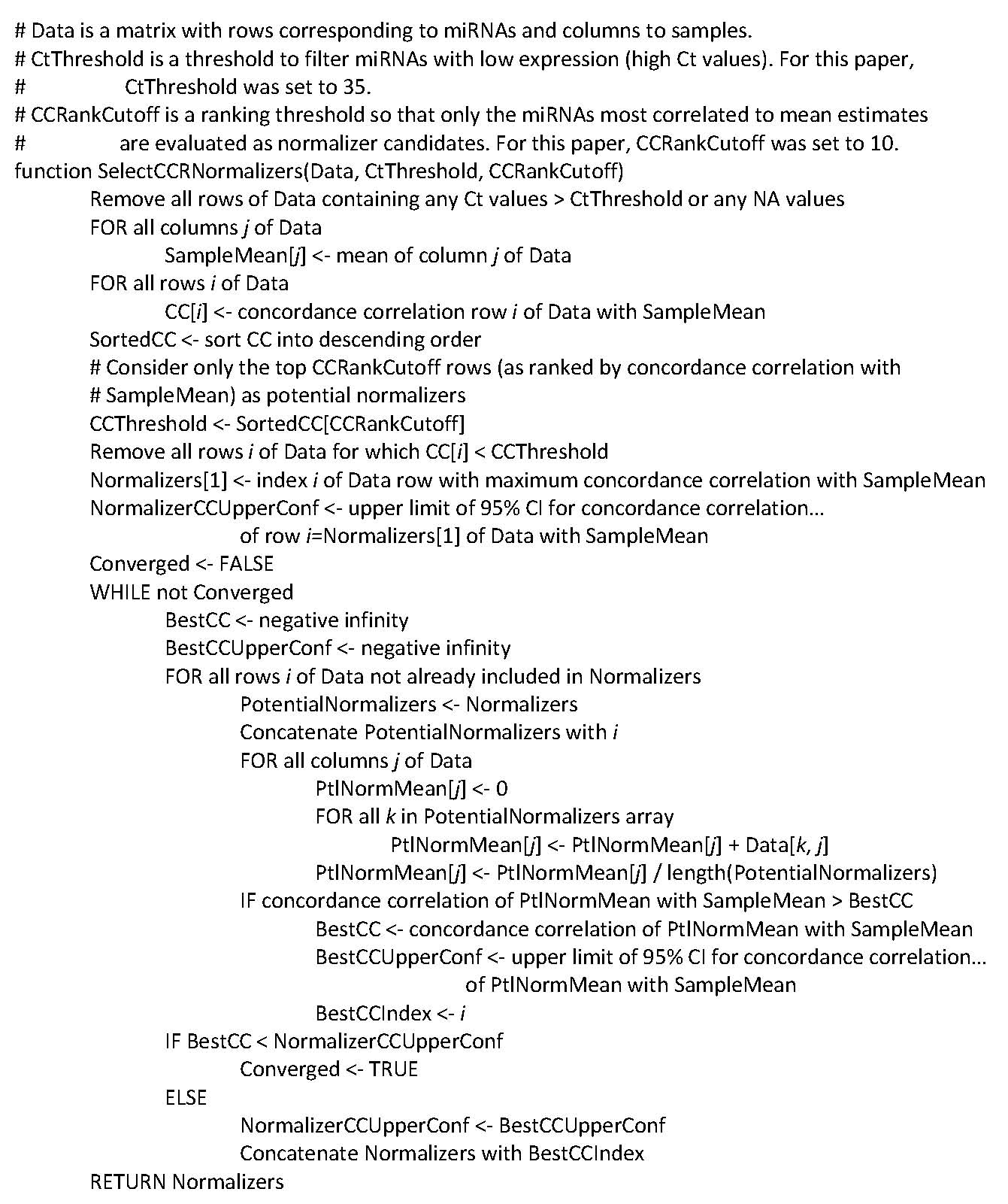


## Supplemental Figure 2 - Pseudocode for the CCR algorithm.

The text details a function, SelectCCRNormalizers, that implements the CCR algorithm. The inputs include the miRNA expression data (Data), a Ct threshold (CtThreshold) for filtering out poor normalizer candidates, and a ranking threshold (CCRankCutoff) for selecting likely candidates. This function is useful for migrating signatures from Megaplex to singleplex RT-qPCR, as the CCR candidates capture the mean estimates established by MCR. For the purposes of this manuscript, we bypassed the WHILE loop and enforced the selection of two miRNA normalizers. This constraint was imposed such that all normalizer selection methods select the same number of miRNA normalizers. In our experience, the CCR estimate usually terminates with 3 or fewer normalizers, and the runtime performance is significantly faster than that of GeNorm and NormFinder (data not shown).


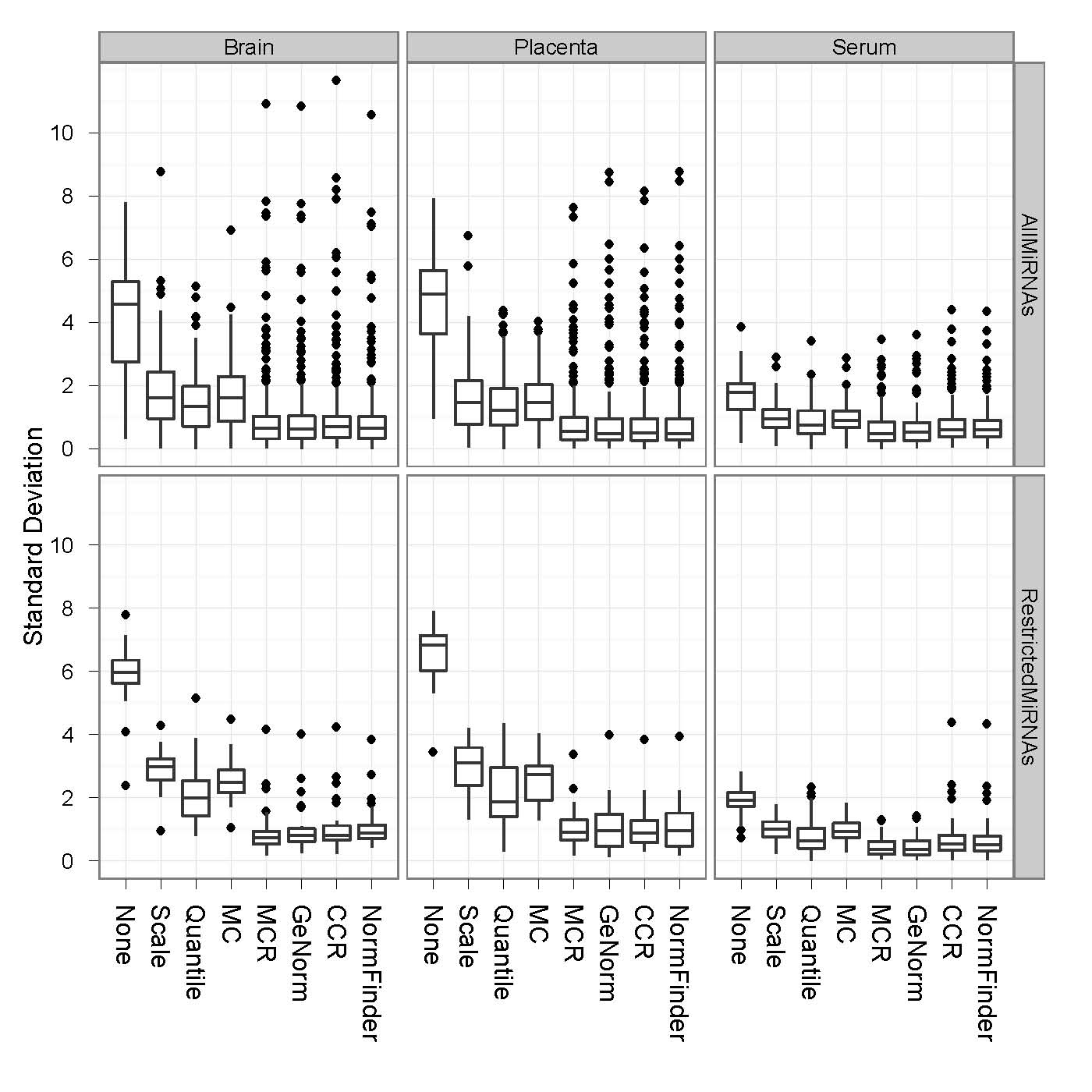


## Supplemental Figure 3 - Distribution of standard deviations of miRNA expression.

Distribution of standard deviations of all miRNAs (top panels) and the restricted set of miRNAs (bottom panels) are stratified by the origin of the samples. When calculating the standard deviations for all miRNAs (AllMiRNAs), non-determined calls (Ct>40) were excluded. **Figure 2** in the main text summarizes these values using a mean estimate.
